# Biomarker-based diagnosis of pacemaker and implantable cardioverter defibrillator pocket infections: A prospective, multicentre, case-control evaluation

**DOI:** 10.1371/journal.pone.0172384

**Published:** 2017-03-06

**Authors:** Carsten Lennerz, Hrvoje Vrazic, Bernhard Haller, Siegmund Braun, Tobias Petzold, Ilka Ott, Agnes Lennerz, Jonathan Michel, Patrick Blažek, Isabel Deisenhofer, Peter Whittaker, Christof Kolb

**Affiliations:** 1 Klinik für Herz- und Kreislauferkrankungen, Abteilung für Elektrophysiologie, Deutsches Herzzentrum München, Technische Universität München, Munich, Germany; 2 Department of Internal Medicine, Division of Cardiology, University Hospital Dubrava, School of Medicine, University of Zagreb, Zagreb, Croatia; 3 Institut für Medizinische Statistik und Epidemiologie, Klinikum rechts der Isar, Technische Universität München, Munich, Germany; 4 Institut für Laboratoriumsmedizin, Deutsches Herzzentrum München, Faculty of Medicine, Technische Universität München, Munich, Germany; 5 Medizinische Klinik und Poliklinik I, Klinikum der Universität München, Munich, Germany; 6 Klinik für Herz- und Kreislauferkrankungen, Deutsches Herzzentrum München, Technische Universität München, Munich, Germany; 7 Cardiovascular Research Institute and Department of Emergency Medicine, Wayne State University School of Medicine, Detroit, United States of America; Kurume University School of Medicine, JAPAN

## Abstract

**Background:**

The use of cardiac implantable electronic devices (CIED) has risen steadily, yet the rate of cardiac device infections (CDI) has disproportionately increased. Amongst all cardiac device infections, the pocket infection is the most challenging diagnosis. Therefore, we aimed to improve diagnosis of such pocket infection by identifying relevant biomarkers.

**Methods:**

We enrolled 25 consecutive patients with invasively and microbiologically confirmed pocket infection. None of the patients had any confounding conditions. Pre-operative levels of 14 biomarkers were compared in infected and control (n = 50) patients. Our selected biomarkers included white blood cell count (WBC), C-reactive protein (CRP), procalcitonin (PCT), lipopolysaccharide binding protein, high-sensitivity C-reactive protein (HS-CRP), polymorphonuclear-elastase, presepsin, various interleukins, tumor necrosis factor α (TNF-α), and granulocyte macrophage colony-stimulating factor (GM-CSF).

**Results:**

Of the 25 patients with isolated pocket infection (70±13years, 76% male, 40% ICDs), none presented with leukocytosis. In contrast, they had higher serum levels of HS-CRP (p = 0.019) and PCT (p = 0.010) than control patients. Median PCT-level was 0.06 ng/mL (IQR 0.03–0.07 ng/mL) in the study group versus 0.03 ng/mL (IQR 0.02–0.04 ng/mL) in controls. An optimized PCT cut-off value of 0.05 ng/mL suggests pocket infection with a sensitivity of 60% and specificity of 82%. In addition TNF-α- and GM-CSF-levels were lower in the study group. Other biomarkers did not differ between groups.

**Conclusion:**

Diagnosis of isolated pocket infections requires clinical awareness, physical examination, evaluation of blood cultures and echocardiography assessment. Nevertheless, measurement of PCT- and HS-CRP-levels can aid diagnosis. However, no conclusion can be drawn from normal WBC-values.

**Clinical trial registration:**

clinicaltrials.gov identifier: NCT01619267

## Introduction

Implantable pacemakers (PM) and cardioverter defibrillators (ICD) are standard treatment for bradyarrhythmias, to ameliorate heart failure, and to protect against sudden cardiac death [1]. Worldwide implantation rates of cardiac implantable electronic devices (CIED) are estimated at 1,250,000 pacemakers and 410,000 cardioverter defibrillators per year, with an annual increase of roughly 5% [2–4]. The recent increase in device utilisation was driven by the needs of an aging population coupled with an expansion of CIED functions and also their indications [5–9]. Nevertheless, increased device use has also increased the incidence of complications. Moreover, these infections have increased disproportionately versus the rate of implantation [2, 10, 11]. This trend in cardiac device infection (CDI) burden is also attributed to the aging population together with an increased prevalence of co-morbidities in current device recipients [2, 5]. The typical estimated annual infection rate is between 1% and 2%; although published rates range from 0.5% to 8% [2, 12–19]. CDI are, not surprisingly, associated with increased costs, significant morbidity, and higher mortality [13, 20–23].

CDI can be classified into three main types; [1] isolated pocket infection, [2] bloodstream infection, or [3] cardiac device related infective endocarditis (CDRIE). Isolated pocket infection is the most frequent, accounting for more than half of CDI [24, 25]. Early diagnosis and subsequent complete device and lead removal, combined with antibiotic treatment, are important to avoid progression to sepsis or endocarditis [5, 16, 26, 27]. However, diagnosing a pocket infection can be challenging because many patients present with few or very mild symptoms or even without any obvious signs of local infection [24, 28–30].

Such frequent lack of clear-cut symptoms of infection place the onus on clinical judgment [28]. Conventional inflammation-related biomarkers, such as white blood count or erythrocyte sedimentation rate, are known to exhibit low sensitivity to cardiac device pocket infections. Hence, they rarely influence diagnostic decisions [24, 31–32]. Therefore, we aimed to evaluate additional inflammatory biomarkers to determine if they enhance diagnosis of cardiac device pocket infection.

## Methods

The DIRT (**D**evice associated **i**nfections–**r**ole of new diagnostic **t**ools) study is a prospective, multicentre, case-control evaluation of inflammatory biomarkers in a cohort of CIED recipients with and without pocket infections. Patients were recruited from five centres in Germany, Croatia, and Italy. From August 2011 to October 2012, consecutive patients with suspected cardiac device pocket infections and control patients at the time of pulse generator exchange or lead revision were invited to participate in the study. The study was approved by the ethical review board of the Technische Universität München, Munich, and conducted according to the principles of the Declaration of Helsinki. The study was registered at clinicaltrials.gov (identifier, NCT01619267). All patients provided written informed consent and 50 mL of peripheral blood were drawn for biomarker analysis.

### Inclusion and exclusion criteria

The inclusion criterion for the evaluation of inflammatory biomarkers was the presence of a cardiac device pocket infection (as described below). Controls were recruited from patients presenting for a pulse generator exchange or lead revision (unrelated to infections) at the same centres during the same period.

Exclusion criteria (for both groups) were the presence of a bloodstream infection or an infective endocarditis; attributed according to modified Duke criteria [33, 34]. Patients were excluded if they presented with current antibiotic treatment, or other concomitant infectious, or inflammatory disease. Additional exclusion criteria were circumstances that could influence inflammatory biomarker levels; e.g. recent trauma, surgery, or burns, patients with active or recent (within two years) malignancy, patients receiving systemic steroid therapy, and patients on high-flux renal dialysis. Minors or adults under guardianship were excluded.

### Diagnostic assessment

**Fig 1:**
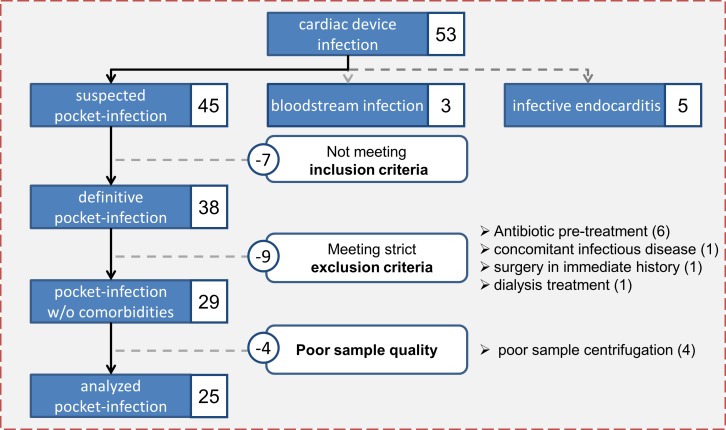
Study flow chart.

**Fig 2:**
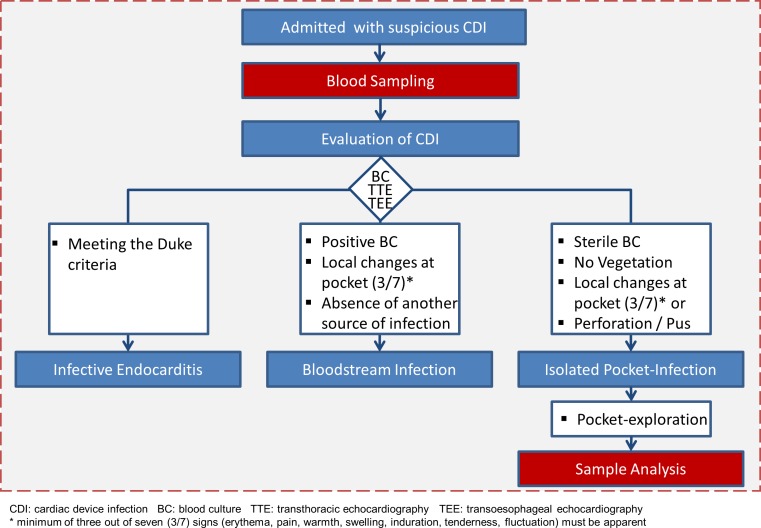
General diagnostic work up and identification of patients with an isolated pocket infection.

Fifty-three patients with cardiac device infection were screened and classified (Fig 1). All patients underwent a baseline assessment, which included; the medical history, the indication for device therapy and device related procedures, physical examination, and basic laboratory tests including white blood count and C-reactive protein (CRP). In all patients with suspected CDI, at least three sets of blood cultures, prior to initiation of antibiotic therapy, were drawn in order to assess potential blood stream infection or infective endocarditis. Additionally, in all these patients, transthoracic and transoesophageal echocardiography was performed to detect valvular vegetation or newly developed valvular insufficiency (Fig 2).

An isolated generator pocket infection was assumed (in the absence of a bloodstream infection or infective endocarditis) if at least three out of the following seven local signs of inflammation or infection were present: erythema, pain, warmth, swelling, induration, tenderness, or fluctuation. Furthermore, hardware protrusion through the skin or pus discharge at the pocket site (either spontaneous or expressed upon palpation of the site) was considered conclusive evidence of pocket infection. In all patients with suspected pocket infection, the diagnosis was required to be confirmed by surgical exploration of the generator pocket site showing purulent or inflammatory changes. In cases of invasively confirmed CDI, we removed all hardware using a transvenous lead extraction approach. In all patients with confirmed CDI, cultures from the device pocket and from the leads were taken for microbiological analysis. If exploration of the pocket failed to reveal a CDI, or if it had not been performed, the patient was ineligible for the study.

After this assessment, patients were classified to have either an infection limited to the generator pocket–representing the target population–or to have an infection with systemic components (i.e., bloodstream infection or definite and possible infectious endocarditis according to the modified Duke criteria) and therefore excluded. [12, 33–34]

### Biomarker selection and analysis

**Fig 3:**
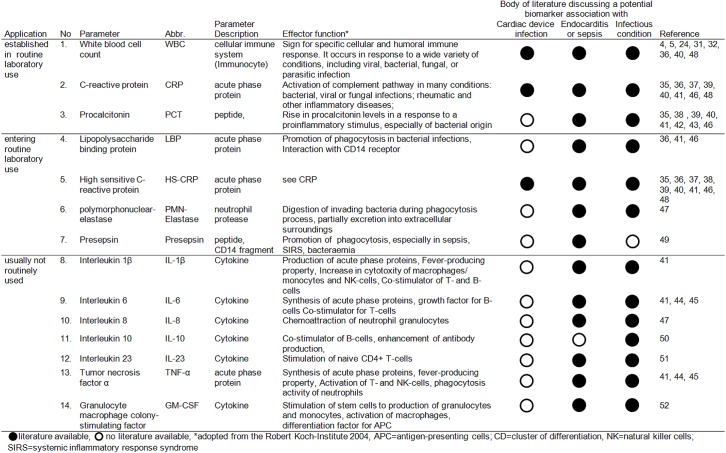
Selection and description of the 14 analysed biomarkers.

In addition to basic laboratory tests of white blood count (WBC) and serum C-reactive protein (CRP), we assessed 12 other biomarkers that could potentially support the diagnosis of cardiac device pocket infection: procalcitonin (PCT), high-sensitivity CRP (HS-CRP), lipopolysaccharide binding protein (LBP), presepsin, polymorphonuclear-elastase (PMN-E), interleukins (IL)-1ß, -6, -8, -10, -23, tumor necrosis factor α (TNFα), and granulocyte macrophage colony-stimulating factor (GM-CSF). The biomarker selection was primarily based on a systematic literature review, searching PubMed with terms related to cardiac device infections and bacterial endocarditis. The query resulted in the identification of 10 biomarkers as promising candidates to detect infection: WBC, CRP, HS-CRP, PCT, LBP, IL-1β, IL-6, IL8, TNF-α and PMN-Elastase (Fig 3) [35–48]. Given the leading causative agents for device infection (bacteria in 80–90% of cases; predominantly coagulase negative staphylococci and staphylococci aureus in 60–70% of cases), and taking into account the inflammatory nature of the process and the specific inflammatory cascades involved, we solicited expert opinion; basic science researchers in immunology or infectious disease and laboratory medicine specialists for their advice on the selection of additional biomarkers for the detection of chronic bacterial infections. Presepsin, IL-10. IL-23 and GM-CSF were the markers suggested by these experts as worthy of evaluation (subsequently substantiated by literature review; Fig 3) [49–52]. The aim of testing numerous biomarkers was to cover all inflammatory process cascades and to identify candidates for subsequent follow-up in a larger study.

After informed consent, and prior to any antibiotic treatment or any invasive procedures, 50 mL of blood were drawn from study participants. The blood samples were divided between heparin, EDTA, citrate, and plasma tubes–according to the assay method–centrifuged, and stored at -70°C. Analysis of biomarker concentrations was performed in a core-lab at the German Heart Centre Munich using commercially available assays (Table 1). All analyses were performed according to the manufacturers´ instructions.

**Table 1:** Detailed overview of applied assays for biomarker-analysis.

Parameter	Abbreviation	Test-Principle*	Assay	Assay manufacturer	Analyser	Analyser manufacturer
White blood cell count	WBC	FFC	Sysmex reagents	Sysmex, Kobe, Japan	XE-5000	Sysmex, Kobe, Japan
C-reactive protein	CRP	TIA	CRP Gen.3	Roche Diagnostics, Mannheim, Germany	cobas c501	Roche Diagnostics, Mannheim, Germany
Procalcitonin	PCT	ECLIA	Elecsys BRAHMS PCT	BRAHMS, Berlin, Germany	cobas e411	Roche Diagnostics, Mannheim, Germany
Lipopolysaccharide binding protein	LBP	CLIA	LBP	Siemens, Erlangen,Germany	Immulite 1000	Siemens, Erlangen, Germany
High-SensitivityC-reactive protein	HS-CRP	CLEIA	Pathfast HS-CRP CLEIA	Progen Biotechnik GmbH, Heidelberg, Germany	Pathfast immunoanalyser	Mitsubishi Chemical Europe, Düsseldorf, Germany
Polymorphonuclear-elastase	PMN-E	ELISA	PMN Elastase (human) ELISA	DRG Instruments GmbH, Marburg, Germany	BEP 2000	Siemens, Erlangen, Germany
Presepsin	Presepsin	CLEIA	Pathfast Presepsin CLEIA	Progen Biotechnik GmbH, Heidelberg, Germany	Pathfast immunoanalyser	Mitsubishi Chemical Europe, Düsseldorf, Germany
Interleukin 1β	IL-1β	CBA	BD CBA inflammatory cytokine kit	BD Biosciences Pharmingen, San Diego, USA	FACS Calibur	BD Biosciences, Heidelberg, Germany
Interleukin 6	IL-6	CBA	BD CBA inflammatory cytokine kit	BD Biosciences Pharmingen, San Diego, USA	FACS Calibur	BD Biosciences, Heidelberg, Germany
Interleukin 8	IL-8	CBA	BD CBA inflammatory cytokine kit	BD Biosciences Pharmingen, San Diego, USA	FACS Calibur	BD Biosciences, Heidelberg, Germany
Interleukin 10	IL-10	CBA	BD CBA inflammatory cytokine kit	BD Biosciences Pharmingen, San Diego, USA	FACS Calibur	BD Biosciences, Heidelberg, Germany
Interleukin 23	IL-23	ELISA	Interleukin-23 (human) ELISA	DRG Instruments GmbH, Marburg, Germany	BEP 2000	Siemens, Erlangen, Germany
Tumor necrosis factor α	TNF-α	CBA	BD CBA inflammatory cytokine kit	BD Biosciences Pharmingen, San Diego, USA	FACS Calibur	BD Biosciences, Heidelberg, Germany
Granulocyte macrophage colony-stimulating factor	GM-CSF	ELISA	GM-CSF (human) ELISA	DRG Instruments GmbH, Marburg, Germany	BEP 2000	Siemens, Erlangen, Germany

* FFC fluorescent flow cytometry, TIA turbidimetric immuno assay, ECLIA electrochemiluminescence immunoassay, CLIA chemiluminescent immunometric assay CLEIA chemiluminescence enzyme immunoassay, ELISA enzyme-linked immunosorbent assay, CBA cytometric bead array

### Endpoint

The study endpoint was to assess the accuracy of 14 biomarkers in diagnosis of cardiac device infection limited to the generator pocket.

### Statistics

The DIRT study was designed as an explorative evaluation of the diagnostic accuracy of biomarkers associated with cardiac device pocket infections. For this, patients were prospectively and consecutively included until the target population—patients with isolated pocket infection naïve to antibiotic pre-treatment—reached 25 and the control group reached 50. Because there was no current data to indicate the potential efficacy of these biomarkers, our study was intended as a preliminary evaluation and hence no pre-investigation power-analysis was performed. Nevertheless, our predefined sample sizes were considered large enough to provide sufficient discrimination.

Categorical data are presented as absolute and relative frequencies, continuous data as mean ± standard deviation or as median with interquartile range (IQR). Comparisons were performed using either chi-square or Fisher exact tests for categorical variables as appropriate. Continuous variables were analysed using a two-sample t-test if normally distributed. Otherwise, the Mann Whitney U-test was used. Initially, for inflammatory biomarkers with established cut-off values, the diagnostic accuracy for isolated pocket infection was described by values of sensitivity and specificity. Subsequently, receiver operating characteristic (ROC) curves were drawn for all biomarkers. The area under the ROC curve (AUC) with 95% confidence intervals was then calculated. Finally, the optimal cut-off value of each biomarker (i.e., the maximized sum of sensitivity and specificity; Youden index) was derived. Statistical analyses were performed using SPSS V.21.0 (IBM Corporation, Armonk, USA).

## Results

### Study samples

According to the modified Duke criteria, cardiac device related infective endocarditis (CDRIE) was diagnosed in five patients, bloodstream infection was confirmed in three, and pocket infection was suspected in 45. After application of the inclusion and exclusion criteria, we analyzed blood samples from 25 patients with invasively confirmed pocket infections (Fig 1).

### Baseline characteristics

Demographic and baseline characteristics are summarized in Table 2. Generally, these did not differ between groups. However, there was weak evidence to suggest that infected patients were more likely to have ischemic cardiomyopathy and to have undergone more CIED procedures more recently. Swab- or lead-cultures were available in 24 out of 25 of patients and were positive in 20 out of 24 (83%) individuals. Pathogens in patients with isolated pocket infection without antibiotic pretreatment included: Staphylococcus epidermidis (n = 12, 50%), Staphylococcus capitis (n = 4, 17%), Staphylococcus aureus (n = 2, 8%), Staphylococcus haemolyticus (n = 1, 4%), Pseudomonas aeruginosa (n = 1, 4%); no specific pathogen was identified in 4 (17%) patients.

**Table 2:** Characteristics of 25 patients with isolated cardiac device pocket infection and 50 control patients.

Characteristic	Study group	Control group	p-value
Number, N	25	50	-
Age [yrs]*	69.8 ± 12.7	69.7 ± 12.6	0.98 ^‡^
Gender, male, N (%)	19 (76%)	33 (66%)	0.38 ^§^
EF, [%]]*	47 ± 13	43 ± 17	0.45 ^||^
Device, ICD, N (%)	10 (40%)	27 (54%)	0.25 ^§^
Creatinine, [mg/dl]]*	1.2 ± 0.6	1.1 ± 0.4	0.48 ^||^
Diabetes mellitus, N (%)	5 (20%)	10 (20%)	1.00 ^§^
Ischemic cardiomyopathy, N (%)	14 (56%)	18 (36%)	0.10 ^§^
Number of CIED-procedures ^†^	1.0	0.8	0.11 ^||^
Months since first CIED-implantation	75	130	0.06 ^||^

* mean ± SD

† exclusive first CIED-implantation

‡ = T-Test

§ = Chi-Quadrat-Test

|| = Mann-Whitney U-Test

EF = ejection fraction; ICD = implantable cardioverter defibrillator; CIED = cardiac implantable electronic devices

### Biomarker evaluation using established cut-offs

None of the routinely used laboratory biomarkers (WBC, CRP, HS-CRP, and PCT) were associated with the presence of pocket infection when established reference values were used. Notably, none of the participants presented with leukocytosis and the serum procalcitonin (PCT)-level never exceeded the upper reference limit of 0.5 ng/mL used in the diagnosis of endocarditis or sepsis. CRP and HS-CRP levels were more often elevated in the study group versus the controls. The difference was more pronounced for HS-CRP; however, the evidence was weak.

In contrast, the less frequently used biomarkers provided stronger evidence of inter-group differences. Presepsin levels above the established cut-off were more prevalent in patients with pocket infections than in healthy controls; the sensitivity and specificity for pocket infection diagnosis was moderate. GM-CSF was found to be significantly more frequently elevated above the reference value in controls than in patients with pocket infections, however, sensitivity and specificity was low. Table 3 provides the details for each of the investigated biomarkers.

**Table 3:** Comparison of biomarker levels between infected and control groups using established cut-offs.

			Study group	Control group			
Biomarker	Unit	Reference value	exceeding reference value N	exceeding reference value N	P-value*	Sensitivity	Specifcity
WBC	*10^9^/l	<10.0	0 / 25	0 / 50	-	0%	100%
CRP	mg/l	< 5.0	8 / 25	7 / 50	0.12	32%	86%
PCT	ng/ml	< 0.5	0 / 25	0 / 50	-	0%	100%
LBP	μg/ml	< 8.4	6 / 25	16 / 50	0.59	24%	68%
HS-CRP	mg/l	< 3.35	11 / 25	10 / 50	0.05	44%	80%
PMN-Elastase	ng/ml	<35.0	20 / 25	33 / 50	0.29	80%	34%
Presepsin	pg/ml	<365	19 / 25	24 / 50	0.03	76%	52%
IL-1β	pg/ml	< 8.0	0 / 25	0 / 50	-	0%	100%
IL-6	pg/ml	< 7.25	4 / 25	4 / 50	0.43	16%	92%
IL-8	pg/ml	<15.0	7 / 25	16 / 50	0.80	28%	68%
IL-10	pg/ml	< 8.0	2 / 25	4 / 50	1.00	8%	92%
IL-23	pg/ml	<15.0	19 / 25	32 / 50	0.43	76%	36%
TNF-α	pg/ml	< 6.0	1 / 25	2 / 50	1.00	4%	96%
GM-CSF	pg/ml	< 0.12	7 / 25	31 / 50	0.01	28%	38%

* Fisher´s exact test.

### Biomarker evaluation using absolute concentrations

**Fig 4:**
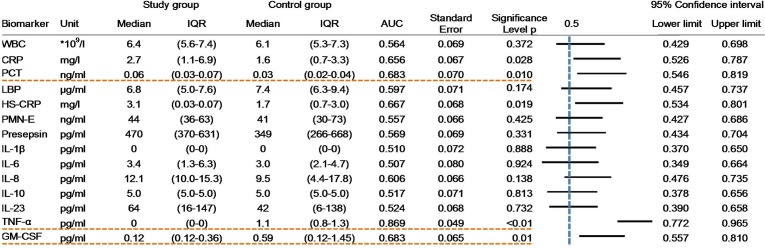
Comparison of biomarker levels between infected and control groups using absolute concentration.

**Fig 5:**
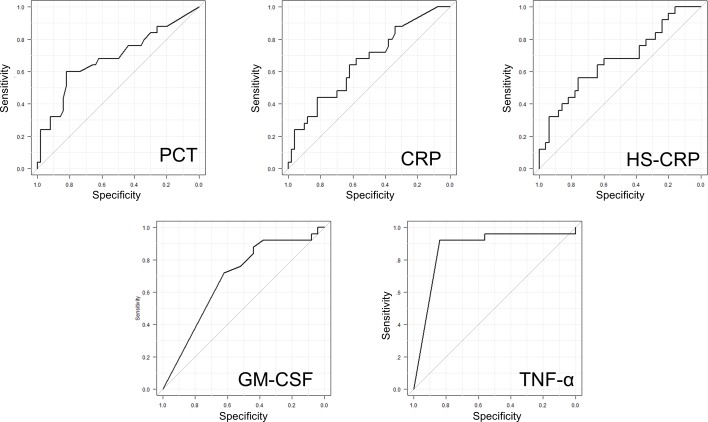
Receiver operator characteristic curve (ROC)-Analysis for the relevant biomarkers.

For each biomarker, the median concentrations and their IQRs and the AUC for each ROC are shown in Fig 4. White blood counts did not differ between groups. However, infected patients had statistically significantly higher serum levels of C-reactive protein (CRP, 2.7mg/L vs. 1.6mg/L, 95% CI 0.53 to 0.79, p = 0.028), high-sensitivity CRP (HS-CRP, 3.1mg/L vs. 1.7mg/L, 95% CI 0.53 to 0.90, p = 0.019), and procalcitonin (PCT, 0.06ng/mL vs. 0.03ng/mL, 95% CI 0.55 to 0.82, p = 0.01) than controls. In contrast, GM-CSF- (0.12pg/mL vs. 0.59pg/mL, 95% CI 0.56 to 0.81, p = 0.01) and TNF-α-levels (0pg/mL vs. 1.1pg/mL, 95% CI 0.77 to 0.97, p<0.01) were lower in infected patients versus controls. The concentrations of presepsin, LBP, PMN-Elastase, and the tested interleukins (IL-1β, IL-6, IL-8, IL-10, IL-23) did not differ between groups. The five biomarkers (CRP, HS-CRP, PCT, TNF-α and GM-CSF) with the largest apparent potential to differentiate pocket infections are illustrated in Fig 5 as ROC curves.

In addition, optimized cut-off values with maximized sensitivity and specificity were obtained from ROC analysis applying the Youden Index. For the biomarkers established in routine diagnostics (CRP, HS-CRP, PCT), the optimized cut-offs resulted in moderate diagnostic power to discriminate between patients with isolated cardiac device pocket infections and healthy controls. Of these, PCT yielded the best combination of sensitivity and specificity. The optimized PCT threshold of 0.05 ng/mL, which is above the 95^th^ percentile of that found in a healthy population, but 10-times lower than the established cut-off for septic conditions, achieved a sensitivity of 60% and specificity of 82% (Table 4). Two of the additional biomarkers tested (TNF-α and GM-CSF), yielded comparable, or somewhat higher, sensitivity and specificity levels with optimized cut-offs. For both markers, low serum concentrations were associated with the presence of infection (Table 4).

**Table 4:** Comparison of sensitivity and specificity for relevant biomarkers applying established or optimized cut-off values.

Biomarker	Unit	established cut-off value	Sensitivity	Specificity	optimized cut-off value	Sensitivity	Specificity
CRP	mg/l	**>5.0**	32%	86%	**>2.1**	64%	62%
HS-CRP	mg/l	**>3.35**	44%	80%	**>3.0**	56%	76%
PCT	ng/ml	**>0.5**	0%	100%	**>0.05**	60%	82%
GM-CSF	pg/ml	**>0.12**	28%	36%	**>0.24**	72%	62%
TNF-α	pg/ml	**>6.0**	4%	96%	**>0.22**	92%	84%

## Discussion

Diagnosis of pocket infections for implanted cardiac devices is challenging and requires considerable experience [35, 48]. Experience is needed first to initiate necessary treatment early enough to avoid development of sepsis or endocarditis. Second, experience is required to avoid unnecessary pocket exploration, with its associated risks, in uninfected patients even when the pocket appears suspicious. Given this spectrum of necessary to unnecessary intervention, biomarkers could aid diagnosis and thereby assist the decision-making process.

In the DIRT study, we provide the first ever assessment of the ability of biomarkers associated with inflammatory processes to differentiate between patients with and without cardiac device pocket infections. Data on such differentiation is scarce and limited to measurement of WBC- and CRP-levels. However, in the majority of patients with cardiac device pocket infection, WBC and CRP levels are normal and therefore provide minimal diagnostic value [24, 32]. These prior observations are confirmed in our study and further underline the unsuitability of non-elevated WBC- and CRP-levels to rule out CDI.

The DIRT study was not restricted to these standard infection parameters, but also assessed 12 additional biomarkers. The group of chosen biomarkers are not specific for the germs causing pocket infections. However, because they cover the different cascades of the inflammatory processes, they represent promising candidates to detect isolated pocket infections. (Fig 3) We used biomarkers with proven responsiveness to bacteria because of the predominately bacterial character of CIED-infection. However, we also included a broad set of interleukins (involved in the specific and unspecific cascade) in order to detect other independent inflammatory processes. This concept of detection of inflammatory reactions is also the same as that underlying the recommended use of F-FDG-PET/CT or SPECT/CT in suspected endocarditis.

When we applied standard cut-offs, increased serum levels of presepsin, and decreased levels GM-CSF, were associated with cardiac device pocket infections. However, the respective sensitivity and specificity provided was, at best, moderate. The finding of increased presepsin serum levels in patients with cardiac device pocket infections appears intuitively reasonable; but, we cannot explain why GM-CSF expression was attenuated.

When we compared the absolute concentration of the biomarkers, we found some evidence of associations for CRP, HS-CRP, PCT, TNF-α and GM-CSF. Increased levels of CRP, HS-CRP and PCT were associated with infections, as were decreased values of TNF-α and GM-CSF. We subsequently used these associations to derive optimized cut-off values and thereby improve the sensitivity and specificity of the tests. However, some of these markers may fail to provide effective and valid screening. For example, CRP concentrations are commonly used to support diagnosis of infective endocarditis and to monitor patient response to therapy [36]. The concentrations tend to be highest in acute S. aureus infections [37], a pathogen also frequently found in cardiac device pocket infections. Application of the optimized reference value to 2.1 mg/L instead of standard 5.0 mg/L increased sensitivity and specificity to 64% and 62%, respectively. However, CRP is not specific for bacterial infections and can also be elevated in viral infections and after surgery or trauma. We excluded these conditions and so evaluation in a less selected patient population could be expected to diminish the apparent differentiating ability. Therefore, CRP does not appear an ideal parameter to detect infection. Similar arguments may also apply to HS-CRP [38]. Furthermore, in terms of sensitivity and specificity, HS-CRP did not provide any additional advantage over the use of CRP.

In contrast, procalcitonin (PCT) serum levels in suspected cardiac device pocket infection may differentiate between healthy and infected patients. Using the optimized cut-off reference value of 0.05 ng/mL (one tenth the standard cut-off value), sensitivity and specificity were 60% and 82%, respectively. This device infection specific PCT cut-off value of >0.05ng/mL is above the normal value in a healthy population. A study of 492 samples (performed with the Elecsys BRAHMS PCT assay) revealed a normal value of 0.046 ng/mL (representing the 95^th^ percentile) [53]. Thus, patients with a PCT value of >0.05ng/mL have less than a 5% chance of a false-positive result, i.e. being a healthy subject.

PCT is known to be an accurate marker for systemic bacterial infection (independent of the pathogen) and, when compared to CRP, it is less prone to influence by viral infections, surgery, or trauma [39]. PCT has also gained importance in the diagnosis and monitoring of infective endocarditis; a differential diagnosis to isolated cardiac device pocket infection. Although PCT levels observed in infective endocarditis differ between assays and also vary according to the duration of infection, they are usually higher than those observed in our cohort of infected patients [35, 40–41]. In our experience, levels of PCT >0.5 ng/mL are typically associated with systemic infection (bloodstream and infectious endocarditis). In order to stimulate future research, we need to prove our pocket-infection-specific cut-off value prospectively. We also need to establish an upper limit that would be suggestive of systemic or septic conditions.

Relatively low–but elevated–serum concentrations of PCT in cardiac device pocket infections may be explained by the localized nature of the infection. In strictly localized infection, there is pronounced increase in PCT levels only if the infection involves surrounding tissues or becomes systemic [42]. Thus, the use of a PCT assay with a low detection limit (in our study 0.02 ng/mL) may be required.

One potential disadvantage of using PCT serum concentration is its rapid decrease in successfully treated patients [42, 43]. Therefore, ideally, blood samples should be drawn before the initiation of antibiotic treatment (as was the case in our study).

The optimized cut-offs for infection diagnosis for GM-CSF and TNF-α produced higher sensitivities and specificities versus CRP, HS-CRP, and PCT. Low serum concentrations of GM-CSF and TNF-α correlated with pocket infection. This finding appears counter-intuitive because, in general, increased expression of these regulators would be expected to occur as part of the inflammatory response. However, these low levels could represent an expression of the entity’s pathogenesis; i.e., a low expression may indicate a poor immune response which thereby facilitates infection. We acknowledge that these explanations are speculative; however, they are hypothesis-generating concepts to stimulate future research. In order to know if and how a cytokine is deregulated in a certain condition, we need to compare normal physiological values with those expressed in the pathology of interest. Curiously, prior studies failed to demonstrate elevated TNF-α-levels in patients with systemic infective endocarditis [44, 45]. The authors speculated that this occurred because of down-regulation of immune cells during persistent stimulation or because of limited ability to stimulate specific cytokines [41].

Another crucial area of research for all relevant biomarkers is to identify the date of applicability after index surgery. From studies analysing WBC count and (18)F-FDG uptake after device implantation/revision, we know the post-operative inflammation process persists for 4–8 weeks after device implantation. For example, after device implantation (<60 days), 10–15% of all patient exhibited a >50% increase in WBC count; a modest WBC count increase of 18 ± 30% was observed for the entire cohort [54]. In addition, Sarrazin et al. reported residual post-operative inflammation present 4 to 8 weeks after surgery [55]. Moreover, the current ESC guidelines on infectious endocarditis recommend a PET/CT scan for infective endocarditis only if the implantation (prosthetic) took place at least three months earlier [56]. Therefore, a conservative approach would be not to use such biomarkers until at least eight weeks after implantation. However, the current use of PCT for stewardship of antibiotic therapy duration in patients after surgery, or with community-acquired pneumonia, may provide a compelling argument that PCT levels respond quickly to changes in inflammatory and infectious conditions. [57, 58]. Also, an in vivo half-life of 22–25 hours provides a rationale for a shorter prohibited period. Nevertheless, we currently lack strong evidence to support the reliability of any biomarker immediately after surgery. However, most patients with suspected pocket infection are admitted several months, rather than weeks, after surgery.

The diagnosis of isolated pocket infection will continue to require a critical clinical awareness, careful patient history assessment, precise physical examination, and a basic workup (i.e. blood cultures, transthoracic and transoesophageal echocardiography). Although one should not draw conclusions from WBC-levels within the normal range, some laboratory parameters (e.g. PCT, CRP, HS-CRP) may provide additional information in the diagnostic decision process. Assessment of PCT has become a routine laboratory parameter used in our evaluation of patients with suspected CIED infection. In cases of possible pocket infection, when the Duke Criteria are not met and local signs are unconvincing (less than three present), we assign a PCT measurement above the cut-off value of 0.05 ng/mL a weight equal to that of a local sign. However, this diagnostic strategy needs to be assessed in a prospective study.

### Limitations

The present study was designed as a pilot evaluation to identify biomarkers worthy of investigation in the diagnosis of cardiac device pocket infections. Therefore, the number of patients included was small. Nonetheless, we assessed a wide variety of biomarkers. CRP, HS-CRP, Procalcitonin, TNF-α, and GM-CSF provided the best discrimination of CDI. However, the specific values used for detection of cardiac device-associated infections differed from standard cut-offs. Our revision of these standard values should be assessed in a prospective study.

Patients with co-morbidities that might interfere with biomarker levels in CDI were excluded. Therefore, our results may some limits to their generalizability. Specifically, our cut-off values may not apply to patients on renal dialysis, patients with altered immune response (e.g., patients on steroids), or after surgery/trauma, or those with concomitant malignant diseases. Furthermore, patients with antibiotic pre-treatment were also excluded because this could influence the results.

## Conclusion

Diagnosis of cardiac device pocket infection remains primarily based on the judgment of experienced physicians. CRP, HS-CRP, and procalcitonin with specific cut-offs for cardiac device infections may provide objective evidence to assist with diagnosis. In contrast, white blood count, lipopolysaccharide binding protein, presepsin, polymorphonuclear-elastase, and interleukins-1ß, -6, -8, -10, -23, do not appear to provide sufficient discrimination to aid diagnosis. The role of depressed levels of tumor necrosis factor α and granulocyte macrophage colony-stimulating factor in cardiac device pocket infections warrants further investigation.

## Supporting information

S1 Trend Checklist TREND statement checklist. (PDF)

S1 Data study data set. (PDF)

S1 Protocol study protocol (English). (PDF)
