# Supplementary material for: Biomarker-based diagnosis of pacemaker and implantable cardioverter defibrillator pocket infections: A prospective, multicentre, case-control evaluation
Source: PLoS One. 2017 Mar 6;12(3):e0172384. doi: 10.1371/journal.pone.0172384 (PMC5338770; doi:10.1371/journal.pone.0172384)
Supplement: S1 Data — (PDF) [file pone.0172384.s002.pdf]

Pat.Nr	Alter	Geschlech	Auswahl	creatinine mg/dl	WBC count in 10^9/l)	CRP in mg/l	PCT	P-SEP in pg/ml	hs CRP in mg/l	PMN-Elastase	GM-CSF	IL-23	IL-6	IL 10	TNF-a pg/ml	IL-8 pg/ml	IL-1ß pg/ml	LBP (µg/ml)
1	75	weiblich	Target	0,88	6,97	0,7	0,054	472	0,622	54,4	0,12	55,99	0,57	22,8	0	6,67	0	4,95
3	71	männlich	Target	0,85	5,25	1,11	0,03	390	0,862	56,13	0,12	30,12	2,13	5	0	11,84	0	5,01
4	42	weiblich	Target	0,54	6,14	7,69	0,02	181	8,1	32,22	0,12	78,55	1,2	5	0	1,86	0	11,2
6	81	männlich	Target	1,12	5,36	2,68	0,056	370	2,35	41,18	0,12	114,5	3,4	5	0	14,64	0	6,78
7	49	männlich	Target	0,95	7,6	0,9	0,04	218	0,641	91	0,12	177,12	1,2	5	0	9,6	0	4,29
8	74	weiblich	Target	0,92	6,4	13,9	0,057	639	3,78	282,05	0,12	109,57	5,07	5	0	19	0	4,86
13	70	weiblich	Target	0,60	4,17	3,78	0,036	419	3,1	34,36	0,36	104,71	0	5	0	10,61	0	2,8
18	72	weiblich	Target	1,13	6,16	8,76	0,054	301	9,65	73,71	0,12	147,14	6,48	5	0	7,8	0	10,7
21	51	männlich	Kontrolle	1,57	5,56	1,28	0,147	339	1,55	40,44	0,12	0,81	1,4	5	0,43	15,65	0	10,4
22	33	männlich	Target	0,83	7,18	0,7	0,022	177	0,533	64,06	6,58	0,54	0,13	5	0	3,49	0	5,46
23	76	männlich	Target	1,02	5,32	3,05	0,026	469	30	26,48	0,12	0,69	39,26	5	0	9,97	0	13,1
25	85	männlich	Target	1,66	6,35	1,98	0,07	415	2,2	46,38	0,12	2,01	4,64	5	0	10,74	0	7,48
26	75	männlich	Kontrolle	1,68	8,49	0,7	0,03	530	0,886	42,95	0,12	203,51	4,68	5	1,56	10,57	0	12,7
27	83	weiblich	Kontrolle	0,69	7,07	2,94	0,047	256	2,90	43,23	0,12	128,35	3,61	5	1,21	7,04	0	7,58
28	65	männlich	Kontrolle	0,84	6,64	0,7	0,034	225	0,335	75,83	1,95	8,94	2,08	5	1,83	3,47	0	7,38
29	83	männlich	Target	3,13	6,99	9,44	0,263	2046	8,68	42,2	13,91	1,44	13,1	5	0	31,49	0	10,7
30	78	männlich	Kontrolle	1,85	6,09	1,85	0,074	1236	1,86	45,51	1,28	0,1	4,68	5	1,1	16,75	0	13,2
31	75	weiblich	Kontrolle	0,63	5,76	1,89	0,028	233	1,88	35,5	0,12	0,31	2,87	5	0,92	3,04	0	7,94
32	62	männlich	Kontrolle	1,30	3,8	7,49	0,043	334	3,22	36,82	0,12	0,45	4,8	5	0,71	12,55	0	12,9
34	67	männlich	Target	1,65	8,81	0,87	0,093	601	1,1	52,38	0,12	60,95	7	5	0	14,31	0	6,99
36	67	männlich	Kontrolle	1,14	4,85	1,23	0,03	241	1,31	24,75	0,36	17,52	2,02	5	0,78	3,88	0	6,98
37	61	männlich	Kontrolle	0,62	7,15	0,82	0,046	359	0,805	29,22	2,4	139,12	1,85	5	7,52	3,69	0	4,61
38	71	männlich	Kontrolle	1,15	7,12	1,11	0,04	455	1,24	31,97	0,12	115,27	3,09	5	0,85	4,77	0	7,02
40	75	männlich	Target	0,96	6,31	2,45	0,105	327	3,06	36,2	0,59	15,68	3,15	5	0	25,22	1,29	6,65
41	70	weiblich	Kontrolle	1,42	5,3	4,19	0,04	1828	4,01	40,44	3,07	0,2	4,12	5	1,03	9,79	0	9,23
42	46	weiblich	Kontrolle	0,53	7,71	0,7	0,026	141	0,297	48,22	27,6	0,38	1,93	5	1,45	5,06	0	6,46
43	80	männlich	Kontrolle	1,26	6,08	3,31	0,02	307	0,847	29,31	0,59	0,11	4,2	5	1,12	4,35	0	7
45	75	männlich	Kontrolle	0,68	3,99	0,7	0,02	246	0,183	24,04	0,12	1,88	1,56	5	1,14	1,33	0	6,69
46	73	männlich	Target	0,91	4,54	3,76	0,056	631	4,1	32,47	0,12	10,58	2,32	5	0	13,34	0	7,55
47	62	männlich	Target	1,07	8,56	6,88	0,024	378	7,23	61	0,12	221,63	5,16	11,8	15,49	15,34	0	6,93
49	71	männlich	Kontrolle	1,38	5,97	2,72	0,02	430	4,36	30,13	0,12	206,07	4,89	5	0	30,35	0	6,94
51	70	männlich	Kontrolle	0,97	7,33	0,91	0,061	290	1,14	87,27	1,5	4,89	2,81	6,05	1,18	19,96	0	10,3
52	65	männlich	Kontrolle	1,21	7,9	1,46	0,064	403	2,47	21,04	7,23	2,71	2,26	5	1,14	4	0	10,1
53	79	weiblich	Kontrolle	0,69	4,55	0,7	0,02	308	0,793	65,87	4,39	0,34	1,66	5	0,81	8,12	0	3,04

54	73	männlich	Target	0,91	6,67	7,74	0,03	473	7,81	41,73	0,59	0,18	4,07	5	0	13,82	0	6,91
55	77	weiblich	Kontrolle	0,78	6,65	1,48	0,036	255	1,8	39,89	0,59	2,56	2,79	21,5	1,21	0,82	0	11,1
56	74	männlich	Target	1,40	6,14	1,63	0,088	470	1,9	30,54	0,12	18,84	1,31	5	0	11,56	0	5,38
57	79	männlich	Kontrolle	0,86	5,76	1,69	0,035	386	1,96	26,72	1,28	9,73	4,23	5	1,56	2,22	0	6,37
58	85	männlich	Kontrolle	1,73	7,69	3,37	0,06	679	3,64	63,63	0,36	107	3,66	5	1,6	30,25	0	9,43
59	87	männlich	Target	1,40	7,5	5,4	0,02	745	15,3	43,99	1,05	47,9	9,33	5	0	21,92	0,49	8,66
61	79	weiblich	Kontrolle	0,69	6,08	0,7	0,029	188	0,491	27,12	1,05	10,58	2,11	5	0,88	2,76	0	4,9
64	70	männlich	Kontrolle	0,82	8,73	0,7	0,024	419	0,92	55,41	1,5	8,94	2,42	5	0,99	4,98	0	4,8
67	81	männlich	Kontrolle	1,83	5,33	8,77	0,056	1098	9,56	34,1	1,05	6,84	5,7	5	0	11,42	0,64	9,16
69	60	weiblich	Kontrolle	0,80	6,6	2,1	0,021	1069	1,15	37,18	1,28	5,67	3,86	5	1,12	7,04	0	12,4
70	52	weiblich	Kontrolle	0,69	4,1	0,7	0,023	273	0,059	21,65	0,59	28,34	2,01	5	1,25	9,41	0	3,25
71	62	männlich	Kontrolle	1,10	7,6	1,52	0,04	308	1,3	23,03	5,27	234,82	2,23	5	1,43	2,76	0	5,83
72	83	männlich	Kontrolle	1,65	7,16	6,74	0,033	810	7,07	34,45	1,05	49,09	9,28	5	1,21	17,32	0	7,83
73	64	männlich	Kontrolle	1,20	4,19	15,6	0,077	323	14,1	59,75	1,28	127,15	3,56	16,7	0,76	18,71	0	13,7
74	81	männlich	Kontrolle	1,13	4,61	0,7	0,027	245	0,44	42,67	2,63	21,68	2,26	7,36	0,92	9,54	0	6,27
75	67	männlich	Kontrolle	0,86	6,23	0,7	0,02	229	0,338	40,9	1,28	95,22	3,13	10,3	1,08	30,87	0	3,93
76	81	männlich	Kontrolle	0,79	8,41	0,72	0,02	517	0,56	29,22	0,59	67,02	2,54	5	1,07	4,08	0	6,6
77	50	weiblich	Kontrolle	0,74	6,12	3,45	0,033	339	3,01	26,88	2,85	25,69	2,79	5	1,14	10,04	0	9,96
78	61	männlich	Kontrolle	1,15	5,21	7,63	0,02	182	11,4	29,39	0,36	237,48	9,78	5	1,16	8,12	0	13,3
80	63	männlich	Kontrolle	1,11	1,11	5,09	0,047	869	5,11	39,89	0,36	443,08	4,56	5	0,9	6,54	0	8,11
81	30	weiblich	Kontrolle	0,74	8,39	0,7	0,02	263	0,337	29,22	0,36	416,36	1,63	5	1,29	1,46	0	5,23
82	72	männlich	Kontrolle	1,09	5,78	2,92	0,075	636	2,5	36,64	0,12	198,39	2,26	5	1,5	11,39	0	8,14
83	79	weiblich	Kontrolle	0,93	6,53	2,55	0,021	244	2,16	56,03	7,67	240,15	1,76	5	1,25	4,56	0	7,97
84	72	männlich	Target	1,86	7,4	2,6	0,133	1151	1,02	2103,66	0,12	253,63	4,15	5	0	12,27	0	4,95
86	88	männlich	Kontrolle	1,28	4,8	2,03	0,033	608	1,79	742,92	16,89	87,01	4,64	5	3,39	23,9	1,78	6,31
89	86	weiblich	Kontrolle	0,77	6	3,96	0,032	436	3,93	892,99	1,28	622,35	6,38	5	0	20,08	0	8,37
91	70	männlich	Kontrolle	1,36	9,3	1,78	0,024	339	1,8	368,99	0,12	291,12	5,72	5	6,74	80,89	0	8,26
93	84	männlich	Kontrolle	1,34	7,4	3,2	0,023	748	2,42	735,74	0,12	25,76	5,42	5	0,92	17,9	0	7,48
94	58	männlich	Kontrolle	1,18	7,9	0,12	0,02	5584	0,172	883,19	0,12	37,11	2,54	5	0	17,97	0	5,67
96	82	weiblich	Target	0,95	5,6	1,1	0,057	1060	0,727	179,28	0,12	63,85	2,05	5	0	89,51	0	3,34
97	84	männlich	Kontrolle	1,57	5	7,4	0,021	892	5,75	172,97	0,12	224,52	12,15	5	0	28,95	0	6,64
98	48	männlich	Kontrolle	0,88	6,3	0,9	0,027	2416	0,711	259,54	0,12	132,99	3,18	5	1,33	8,69	0	5,68
99	81	weiblich	Kontrolle	1,08	4,5	3,3	0,02	1785	2,68	209,85	0,12	199,37	5,55	5	1,6	14,96	0	12,9
100	46	männlich	Kontrolle	1,03	5,9	0,3	0,03	305	0,257	352,95	0,12	120,14	0,81	5	0	7,6	0	5,89
101	81	weiblich	Kontrolle	0,88	5,6	0,4	0,04	1234	0,619	348,46	0,12	47,19	15,36	9,5	0	146,6	0	4,75
102	76	weiblich	Kontrolle	2,22	8,3	0,68	0,039	561	0,675	699,37	0,12	99,92	2,29	5	0,92	67,27	0,18	6,93
107	62	männlich	Target	1,39	9,09	27,4	0,097	512	30	62,56	0,86	98,17	6,32	5	1,05	18,56	0	17
108	63	männlich	Target	2,71	7,36	0,7	0,069	544	0,413	40,73	0,12	234,23	1,31	5	0	6,08	0	6,06

110	70	männlich	Target	1,13	6,04	2,12	0,02	324	4,96	37,99	0,12	149,8	9,39	5	0	10,87	0	7,42
120	65	weiblich	Kontrolle	1,15	5,84	3,2	0,068	296	3,28	47,08	0,12	18,72	1,95	5	0	12,72	0	9,42
122	75	männlich	Target	1,09	5,26	3,82	0,06	750	3,23	36,49	0,12	160,88	2,45	5	0	12,13	0	6,2
