# Supplementary material for: Biomarker-based diagnosis of pacemaker and implantable cardioverter defibrillator pocket infections: A prospective, multicentre, case-control evaluation
Source: PLoS One. 2017 Mar 6;12(3):e0172384. doi: 10.1371/journal.pone.0172384 (PMC5338770; doi:10.1371/journal.pone.0172384)
Supplement: S1 Protocol — (PDF) [file pone.0172384.s003.pdf]

7. Prüfplan

DIRT-Study

Device-associated **I**nfections – **R**ole of new diagnostic **T**ools

I. Background:

Implantation of cardiovascular implantable electronic devices (CIED), which include permanent pacemakers (PM) and implantable cardioverter-defibrillators (ICD) has rapidly increased over the last 2 decades.

This trend is largely due to the expanded indications for CIED implantation based on the results of large clinical trials of ICDs for primary prevention as well as the aging of the general population. (Greensporn et al, 2011; Myerburg et al, 2008; Bardy et al, 2005; Moss et al, 2002).

The Greensporn group identified based on the US nationwide inpatient sample (NIS)-database that the incidence of CIED implantation increased on average of 4,7% annually, and the overall CIED implantation increased by 96% from 1993 to 2008. The majority of this increase was due to a large increase in ICD-implantation (504%) as pacemaker implantation increased by 45% over the analyzed time period. (Greensporn et al, 2011)

Device related infections are infrequent but are surprisingly increasing according to the Greensporn analyses. The annual rate of CIED infection remained fairly constant around 1.5% from 1993 until 2004 and then increased significantly to 2.4% in 2008. (Greensporn et al, 2011)

This trend in CIED infection burden is associated with an increasing age and a higher number of clinical comorbidities of the device recipients. (Baddour LM et al, 2010, Greensporn et al, 2011)

An analysis of CIED implantation in the United States between 1997 and 2004 showed that approximately 70% of device recipients were 65 years of age or older, and more than 75% of them had 1 or more coexisting illnesses. (Zhan C, 2008) Furthermore Greensporn observed an increased incidence of 4 major comorbidities (renal failure, respiratory failure, heart failure and diabetes) in patient with CIED infection since 2004. This more comorbid condition of patients coincidenced with the higher rates of CIED infection. (Greensporn et al 2011)

CIED infections are associated with a significant mortality. The early mortality rate (up to 30 days) and the late mortality rate is 2.0% to 15% and 4.8% to 17.5%, respectively, depending on the extent of the CIED infection. (LE K.Y. et al, 2011)

The extent of CIED-infections can be classified in isolated pocket infection, bloodstream infection and CIED-related infective endocarditis (IE). An analysis regarding the clinical presentation of the different types of CIED infection revealed that the most common entity is an isolated pocket infection in one half of the cases (52%), followed by device related endocarditis (23%), pocket

infection with bloodstream infection (17%) and bacteraemia without localizing signs at pocket. (Sohail MR et al, 2007).

The presence of a device related endocarditis is simple to confirm when valvular or lead vegetations are detectable by echocardiography or if the Duke criteria for infective endocarditis are met. CIED bloodstream infections can be generally proved based on positive blood cultures. (detailed definitions of different types of CIED infections see below) Furthermore basic inflammatory parameters, like CRP concentration and the WBC count, are additional useful biomarker leading to the diagnosis of and monitoring a bloodstream infection or IE.

Confirming the diagnosis of an isolated pocket infection without bacteraemia, which is by far the most frequent CIED infection (> 50% of all CIED infection), is a much more challenging. By definition of the entity echocardiography and microbiological analysis cannot lead to the diagnosis. In addition an isolated pocket infection is infrequent associated with an elevation of basic inflammatory markers. Sohail et al. found that only 43% of all CIED infection goes along with a leucocytosis (WBC >10000/µl) and only 25% of all CIED infection show a high erythrocyte sedimentation rate (ESR > 22mm/h in men and > 29mm/h in women). (Sohail et al, 2007) Assuming that the inflammatory markers are more frequent elevated in CIED infections going along with bacteraemia and endocarditis the respective percentage is even lower. It remains to find the diagnosis of an isolated pocket infection based on the clinical presentation. Local signs of inflammation at the generator pocket, including erythema, pain, warmth, swelling, induration, tenderness, fluctuation, perforation, purulent discharge can support the diagnosis.

However typical clinical signs and symptoms are often lacking and consequently the diagnosis of a pocket infection is even more demanding. Sohail et al described local findings at the generator site with a frequency of 25% to 68%. Chamis et al. even found that in 60% of confirmed CIED-infections in presence of a positive pocket side and blood culture, no local signs or symptoms suggesting a pocket infection were noted. (Chamis A.L. et al; 2001)

Due to that frequently oligosymptomatic or clinically inapparent course the discrimination between an isolated pocket infection and relevant differential diagnosis, as an imminent perforation of the skin or decubital gangrene is not only challenging but also crucial regarding the therapeutic conclusions.

Complete device and lead removal is recommended for all patients with a CIED pocket infection (Epstein et al. 2010), whereas pocket complication with intact skin can be treated successfully with surgical revision.

Regarding the outcome and the therapeutic management the accurate identification of CIED is essential to ensure that patients with infection involving a permanent pacemaker or implantable cardioverter-defibrillator are treated appropriately and that those patients with uninfected devices avoid unnecessary removal. (Chamis A.L. et al, 2001)

Aim of the study is to identify new supportive biomarker for the diagnosis and therapeutic monitoring of CIED-infection, especially of isolated pocket infection.

II. Hypothesis:

Several biomarkers can be considered as diagnostic tools to identify device related infections. The most promising candidates are:

1) Pro-Calcitonin (PCT)

Pro-Calcitonin is an established biomarker for bacterial infection, especially with a diagnostic value in sepsis. Furthermore PCT is known for a higher diagnostic accuracy than CRP in differentiating bacterial from non-infective causes of inflammation. Hence Pro-Calcitonin is a potential candidate to bridge the diagnostic gap in isolated pocket infections.

2) Lipopolysaccharide-binding protein (LBP)

Lipopolysaccharide-binding protein has recently been described as a novel diagnostic marker for the diagnosis of local bacterial infection. The production of LBP takes place predominantly in the hepatocytes and the release occurs upon acute-phase stimulation. Several studies have shown that there are increased serum levels of LBP due to gram-positive, gram-negative and fungal infection. An association with viral infection has not been observed yet. Several studies have reported LBP as a valuable biomarker for acute gastrointestinal infections, for severe sepsis and for infectious endocarditis. (Vollmer T et al, 2009; Mierzchala M et al, 2001)

3) PMN-Elastase

PMN-Elastase is an established biomarker for chronical inflammation and a diagnostic marker in sepsis. The PMN Elastase is released from neutrophil granulocytes after irritation or disintegration. A bacterial infection goes along with an increase of increased phagocytic activity and the decay of the cells leads to an increased release of PMN-Elastase. These days PMN-Elastase is an diagnostic tool for identification and monitoring of chronic inflammation diseases of the gut (Crohn´s disease and ulcerative colitis), chronic joint inflammation as well as bacterial infection and sepsis. (Endo S et al, 1995)

4) Interleukin (IL1, IL6, IL8, IL10, IL12, TNFα)

As any immune response is mediated by interleukins, it makes sense to evaluate the clinical usefulness of these cytokines as an indicator for CIED infections, especially for the isolated pocket infection. The chosen set of interleukins (IL1, IL6, IL8, IL10, IL12, TNFα) can reveal a specific immune response (T-helper cell type 1 and type 2 mediated) as well as an unspecific immune response (macrophage cells, monocytes and neutrophil granulocytes regulated) to the underlying bacterial infection. Several studies have investigated the application and usefulness of interleukins for IE diagnosis (Vollmer T et al, 2009), but there are no reports about the diagnostic value of interleukins in the other entities of CIED infections.

5) Granulocyte macrophage colony-stimulating factor (GM CS-Factor) and Interferon-γ (IFN-γ)

Both cytokines are part of the immune or inflammatory cascade, by which macrophage cells are activated and increase in their number. This process is crucial for fighting bacterial infection. Elevated levels in CIED infections can probably used as a diagnostic tool.

III. Study design:

DIRT-study is designed as a controlled, multicenter, prospective pilot study.

IV. Inclusion and Exclusion criteria

- Inclusion Criteria

  Consecutive patients with a CIED-infection disregarded the extent of the infection (i.e. isolated pocket-, bloodstream infection or CIED-related infective endocarditis; definitions see below) are included.

- Exclusion Criteria

  Patients may not be considered for the study if they exhibit any of the following

  1) concomitant infectious disease unrelated to the CIED-infection
  2) trauma, surgery or burns in the immediate history
  3) Active or recent malignancy within two years of curative treatment without evidence of recurrence
  4) high dose steroid therapy (more than 40 mg daily)
  5) Minors or patients under tutelage

V. Definitions:

Due to extent of the infection we distinguish three different entities of CIED infections: an isolated pocket infection, a bloodstream infection and a CIED-related infective endocarditis (IE). Thereby we made use of definitions described by other groups. (adapted from Sohail et al, 2007, Durack DT et al, 1994, Ohlow M.-A. et al, 2001)

1) Isolated pocket infection

   Only definitive pocket infections were included. Definitive pocket infection was confirmed by

   - purulent discharge at the pocket side, either spontaneous or expressed upon palpation of the site, regardless of whether an organism is cultured from the pocket site
   - purulent discharge at surgical exploration of the generator pocket, regardless of whether an organism is cultured from the pocket site

- local signs of inflammation or infection at the generator pocket (any three of the nine: erythema, pain, warmth, swelling, induration, tenderness, fluctuation, perforation, positive culture from the pocket)

2) CIED bloodstream infection
Device related bloodstream infection was microbiologically confirmed based on positive blood cultures in the presence of local inflammatory signs at generator pocket or absence of another source of bacteraemia and resolution of blood stream infection after device explantation.

3) CIED related infective endocarditis (IE)
Device related endocarditis was clinically confirmed when valvular or lead vegetations were detected by echocardiography, or if the Duke criteria for infective endocarditis were met. Clinical criteria for infective endocarditis required two major criteria or one major and three minor criteria or five minor criteria. Vegetation was defined as an oscillating intracardiac mass on the electrode leads, cardiac valve leaflets, or endocardial surface in the setting of valve or lead infection confirmed by imaging in more than 1 echocardiographic plane, and positive blood and/or lead tip cultures)

VI. Study procedure

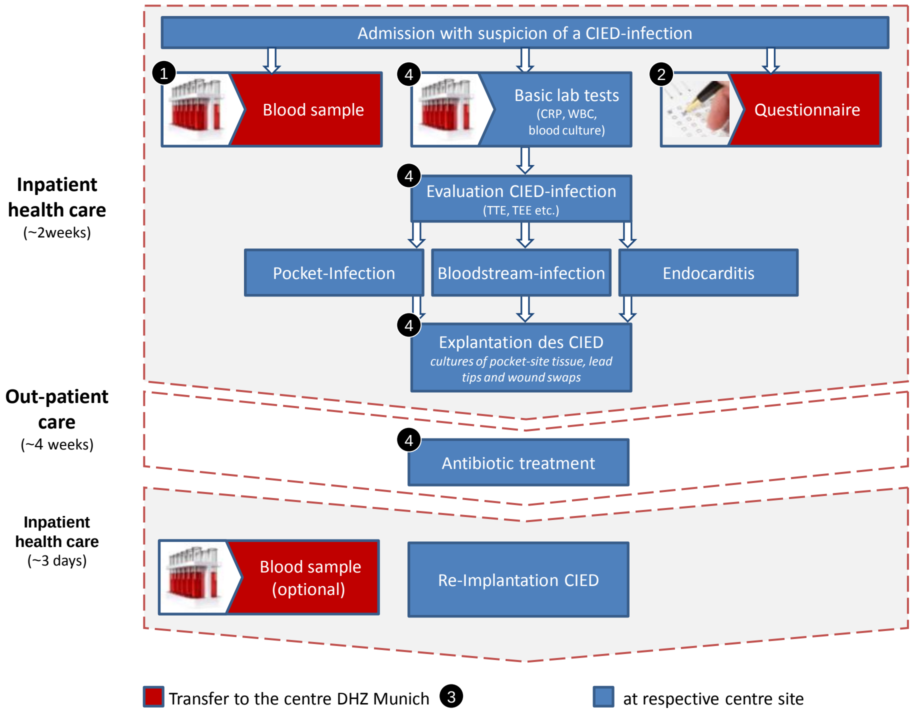

Action needed by the participating centers:

1) Allocation of the defined venous blood samples after inclusion
2) Answering a detailed questionnaire regarding the extend of CIED-infection, the signs and symptoms on admission, the underlying cardiac disease, the cardiac device history, the antimicrobial- and interventional /surgical-management and the follow-up
3) transfer of centrifuged samples and questionnaire to the single processing center (DHZ München)
4) Executing of diagnostic (blood cultures, trans-thoracic and trans-oesophageal echocardiography, basic lab tests (CRP, WBC), cultures of pocket-site tissue, lead tips and wound swaps) as well as therapeutic-standards (antimicrobial therapy, removal of infected CIEDs) are mandatory.

Action needed at the processing centre DHZ Munich

1) Laboratory processing of the all transferred blood samples

VII. Endpoints

1) Diagnostic role of PCT in isolated pocket infection:
   PCT-levels in isolated pocket infection compared to a control who present with implanted devices for device exchange or revision
2) Diagnostic role of PCT in differentiation between different entities of CIED infection:
   PCT-levels in isolated pocket infections compared to PCT-levels in patients with blood stream infections or endocarditis
3) Diagnostic role of Lipopolysaccharide-Binding Protein (LBP) in different conditions of CEID infection (analysis as in 1) and 2))
4) Identification of other biomarker among interleukins, TNF-α, high sensitivity C-reactive Protein (HS-CRP), Interferon-γ, PMN-Elastase and GM-CSF etc., that may better help to diagnose or differentiate the different extents of CIED-infections (by analogy with 1) and 2))
5) Evaluate if any of the above mentioned biomarkers would be suitable to monitor the treatment success of isolated pocket infections:
   - Comparison of biomarker concentrations between patients native to antibiotic treatment with patient who already received empirical and suitable antibiotics at the time of admission.
   - Comparison of biomarker level under treatment with empirical or suitable antibiotics

VIII. Study related risks / complications

In suspicion of a CIED infection the diagnostic guidelines recommend taking a blood sample and attaining a blood culture in order to reveal an infection or a bacteraemia anyway. Together with that routine diagnostic blood sample we obtain an additional blood volume of 50 ml for the study purposes. From a medical perspective this extra loss of blood is negligible and will not harm the subjects.

The data acquisition is completely anonymous. Thus there is neither potential risk for the patients in participating in the study nor is there any benefit for the participating individual.

IX. Setting of the study

In order to include a reasonable number of patients at each of the centres, only high volume centres of CEID implantation will be invited to participate as only such centres will have enough infections occurring at their site or will have enough patients referred for the treatment of CEID associated infection.

Planned investigational sites are by now

- three University Hospital:
  1) Department of Cardiovascular Diseases, German Heart Centre Munich / 1st Medical and Outpatients Department of Rechts der Isar Hospital, Technical University of Munich, Germany
  2) Cardiology Division, Department of Internal Medicine, University Hospital Dubrava, Zagreb, Croatia
  3) Cardiology Department Ain Shams University, Cairo, Egypt and
- several non-universitary centre with a high implantation volume
  1) 1st Medical Department, hospital Landshut-Achdorf
  2) Cardiology Department, Dunant Hospital, Athens, Greece

X. Statistics and number of cases

The study planned as a multicenter, prospective, controlled pilot study. The aim of the study is to identify new biomarkers as a tool in order to diagnose oligosymptomatic or clinical inapparent pocket infections. This pilot study is designed to investigate different promising candidates (i.e. Procalcitonin, Lipopolysaccharide binding protein, different interleukine etc) and to generate further hypothesis based on the findings, which are to verify in continuative studies.

Therefore, consecutive patients with CEID infection will be included in every centre until the target population of patients with proved isolated pocket infections not on antibiotic pre-treatment reaches 25 patients. According to the distribution of referred patients it is expected to recruit a pool of patients with pocket infections who already received antibiotics on the inclusion and patients with bloodstream infections or device related endocarditis.
A control group of 100 patients with implanted CEID and no signs of infection (e.g. at the time of battery exchange or lead revision) will be used as controls in a matched pair configuration. International centres will contribute to the control patient pool. Matching will be done according to age, gender, underlying cardiac disease (ischemic versus non-ischemic) and comorbidity (e.g. diabetes mellitus etc.), as well LV ejection fraction.

XI. Study start:

Planned after ethics committee approval 12/2011

XII. Principle investigators:

PD Dr. med. C. Kolb, Klinik für Herz- und Kreislauferkrankungen, Deutsches Herzzentrum München
Dr. med. C. Lennerz, Klinik für Herz- und Kreislauferkrankungen, Deutsches Herzzentrum München

XIII. References

1) Baddour LM, Epstein AE, Erickson CC, Knight BP, Levison ME, Lockhart PB, Masoudi FA, Okum EJ, Wilson WR, Beerman LB, Bolger AF, Estes NA 3rd, Gewitz M, Newburger JW, Schron EB, Taubert KA, Update on cardiovascular implantable electronic device infections and their management: a scientific statement from the American Heart Association. Circulation. 2010 Jan 26;121(3):458-77. Epub 2010 Jan 4.
2) Bardy GH, Lee KL, Mark DB, et al. Sudden cardiac death in heart failure trial (SCD-HeFT). Amiodarone or an implantable defibrillator for congestive heart failure. N Engl J Med 2005;352:225–37.
3) Chamis AL, Peterson GE, Cabell CH, Corey GR, Sorrentino RA, Greenfield RA, Ryan T, Reller LB, Fowler VG Jr.; Staphylococcus aureus bacteremia in patients with permanent pacemakers or implantable cardioverter-defibrillators. Circulation. 2001 Aug 28;104(9):1029-33.
4) Durack DT, Lukes AS, Bright DK. New criteria for diagnosis of infective endocarditis: utilization of specific echocardiographic findings: Duke Endocarditis Service. *Am J Med*. 96:200-209, 1994.

5) Greenspon AJ, Patel JD, Lau E, Ochoa JA, Frisch DR, Ho RT, Pavri BB, Kurtz SM., 16-year trends in the infection burden for pacemakers and implantable cardioverter-defibrillators in the United States 1993 to 2008. J Am Coll Cardiol. 2011 Aug 30; 58(10):1001-6.
6) Endo S, Inada K, Ceska M, Takakuwa T, Yamada Y, Nakae H, Kasai T, Yamashita H, Taki K, Yoshida M., Plasma interleukin 8 and polymorphonuclear leukocyte elastase concentrations in patients with septic shock. J Inflamm. 1995;45(2):136-42.
7) Le KY, Sohail MR, Friedman PA, Uslan DZ, Cha SS, Hayes DL, Wilson WR, Steckelberg JM, Baddour LM; Mayo Cardiovascular Infections Study Group, Impact of timing of device removal on mortality in patients with cardiovascular implantable electronic device infections. Heart Rhythm. 2011 May 27. [Epub ahead of print]
8) Mierzchala M, Krzystek-Korpacka M, Gamian A, Durek G. Quantitative indices of dynamics in concentrations of lipopolysaccharide-binding protein (LBP) as prognostic factors in severe sepsis/septic shock patients--comparison with CRP and procalcitonin. Clin Biochem. 2011 Apr;44(5-6):357-63. Epub 2011 Feb 1.
9) Moss AJ, Zareba W, Hall WJ, et al., for the Multicenter Automatic Defibrillator Implantation Trial II Investigators. Prophylactic implantationof a defibrillator in patients with myocardial infarction and reduced ejection fraction. N Engl J Med 2002;346:877– 83.
10) Myerburg RJ. Implantable cardioverter-defibrillators after myocardial infarction. N Engl J Med 2008;359:2245–53.
11) Ohlow MA, Lauer B, Buchter B, Schreiber M, Geller JC, Pocket related complications in 163 patients receiving anticoagulation or dual antiplatelet therapy: D-Stat Hemostat™ versus standard of care. Int J Cardiol. 2011 Mar 3. [Epub ahead of print]
12) Sohail MR, Uslan DZ, Khan AH, Friedman PA, Hayes DL, Wilson WR, Steckelberg JM, Stoner S, Baddour LM. Management and outcome of permanent pacemaker and implantable cardioverter-defibrillator infections. J Am Coll Cardiol. 2007 May 8;49(18):1851-9. Epub 2007 Apr 23.
13) Vollmer T, Piper C, Kleesiek K, Dreier J., Lipopolysaccharide-binding protein: a new biomarker for infectious endocarditis? Clin Chem. 2009 Feb;55(2):295-304. Epub 2008 Oct 2.
14) Zhan C, Baine WB, Sedrakyan A, Steiner C, Cardiac device implantation in the United States from 1997 through 2004: a population-based analysis. J Gen Intern Med. 2008 Jan;23 Suppl 1:13-9.
